# Fabrication and evaluation of BerNPs regarding the growth and development of *Streptococcus mutans*

**DOI:** 10.3762/bjnano.16.23

**Published:** 2025-02-27

**Authors:** Tuyen Huu Nguyen, Hong Thanh Pham, Kieu Kim Thanh Nguyen, Loan Hong Ngo, Anh Ngoc Tuan Mai, Thu Hoang Anh Lam, Ngan Thi Kim Phan, Dung Tien Pham, Duong Thuy Hoang, Thuc Dong Nguyen, Lien Thi Xuan Truong

**Affiliations:** 1 Laboratory of Biotechnology, Research Laboratories of Saigon Hi-Tech Park, Ho Chi Minh City, Vietnam; 2 Faculty of Medical Technology, Van Lang University, Ho Chi Minh City, Vietnam; 3 Department of Biotechnology, Nong Lam University, Ho Chi Minh City, Vietnam; 4 Faculty of Pharmacy, Van Lang University, Ho Chi Minh City, Vietnam

**Keywords:** antibacterial, berberine nanoparticles, BerNPs, biofilm, FE-SEM, *Streptococcus mutans*

## Abstract

In this study, berberine nanoparticles (BerNPs) were prepared using a wet-milling method with zirconium balls to enhance bioavailability and expand potential applications. The particle size and physicochemical properties of the BerNPs were analyzed using field-emission scanning electron microscopy (FE-SEM), UV–vis spectroscopy, X-ray diffraction, and Fourier-transform infrared spectroscopy. The broth dilution method was used to determine the antimicrobial activity of the BerNPs against *Streptococcus mutans* (*S. mutans*). The impact of the BerNPs on the cell surface of *S. mutans* was evaluated through FE-SEM analysis, focusing on its ability to inhibit biofilm formation. The results demonstrated that BerNPs were produced with an average particle size of 40–65 nm. The chemical structure of BerNPs remained consistent with that of berberine, with no modifications occurring during nanoparticle preparation. The BerNPs exhibited the ability to inhibit *S. mutans*, with minimum inhibitory concentration and minimum bactericidal concentration values of 78.1 and 312.5 µg/mL, respectively. BerNPs caused significant damage to *S. mutans* cells, disrupting the cell membrane structure, and leading to cell lysis and death. Additionally, BerNPs effectively inhibited the biofilm formation of *S. mutans*. In summary, BerNPs demonstrated a potent inhibitory effect on the activities of *S. mutans* at selectively applied concentrations.

## Introduction

According to the Global Burden of Disease study, the global prevalence of oral diseases increased from 2.5 billion individuals in 1990 to 3.5 billion individuals in 2017 [1]. Of these, approximately 2.5 billion individuals suffer from chronic tooth decay. In Vietnam, the prevalence of these diseases is notably high, with up to 90% of the population affected by dental disease, including 85% of children who do not fully recover [2]. Tooth decay is a chronic condition that can occur at any age, primarily caused by an improper diet and inadequate oral care, which promote the proliferation of pathogenic bacteria in the mouth [3]. Furthermore, the progression of tooth decay is directly related to the ability of bacteria to penetrate the tooth surface and form plaque. A combination of bacteria in dental plaque causes tooth decay. Among the more than 700 species of bacteria in dental plaque, most belong to the genus *Streptococcus*. Research on dental plaque from various ethnic groups worldwide indicates that 70–100% of strains belong to the group of *S. mutans*. Numerous studies have also identified this bacterium as the primary cause of tooth decay. Consequently, *S. mutans* is frequently used as a model organism in studies on dental caries [4–5]. *S. mutans* can ferment carbohydrates, primarily sucrose and glucose or by-products in saliva, to produce weak organic acids. These acids lead to tooth surface demineralization and the subsequent enamel loss, resulting in tooth decay [6–7]. Furthermore, the biofilm formation by *S. mutans* contributes to plaque formation associated with caries damage. Therefore, one of the initial steps in preventing dental caries is to reduce and inhibit the activity of *S. mutans* in the oral cavity [8]. Tooth decay and oral infections are typically controlled with antibiotics. However, the proliferation of drug-resistant bacteria and the associated adverse effects, such as allergies, have increased the prevalence of using natural compounds to treat bacterial diseases. Among these, berberine, a plant-based alkaloid traditionally used in medicine, has been recognized for its antibacterial, antiviral, and anti-inflammatory properties [9]. With its antibacterial activity, berberine can accumulate in bacterial cells and bind to single- and double-stranded DNA, causing DNA damage. According to evaluations, berberine has a stronger antibacterial effect against gram-positive bacteria than against gram-negative bacteria. It also exhibits antifungal activity against *Aspergillus*, *Penicillium*, *Candida*, and *Cryptococcus* [10]. While having several benefits, berberine has limited therapeutic usage since it is poorly soluble in water, absorbs poorly through the intestinal wall, and has a very low bioavailability (about 5%) [11]. Therefore, nanotechnology has been applied to solve this problem.

The physical, chemical, and biological properties of nanoscale materials are significantly different from those of their larger counterparts. As a result, numerous studies have been conducted to apply nanotechnology to obtain materials with novel properties. The development of substances and methods with enhanced water dispersibility and bioavailability from materials such as berberine and curcumin is a current trend. Several studies on the nanofabrication of berberine aimed at improving its bioavailability and evaluating its inhibitory activity against pathogenic bacteria have been reported. In 2022, Nguyen et al. fabricated berberine nanoparticles (BerNPs) by antisolvent precipitation (ASP) using glycerol as a safe organic solvent and evaluated their antibacterial activity on *S. aureus* and *E. coli* O157:H7 [12]. Additionally, several methods of berberine encapsulation, berberine carrier materials, and berberine microemulsions have also been studied to improve the bioavailability and clinical applicability of berberine [9,13–17].

Although many studies have evaluated the antibacterial activity of berberine, there is currently little information on its antimicrobial activity against *S. mutans*. Therefore, it is scientifically and economically important to clarify the mechanism and ability of BerNPs to inhibit the growth of *S. mutans*. The findings from such studies could serve as a foundation for developing products containing BerNPs, which can be used in the care and treatment of dental caries and other dental problems.

## Results and Discussion

### Production of BerNPs

**Figure 1:**
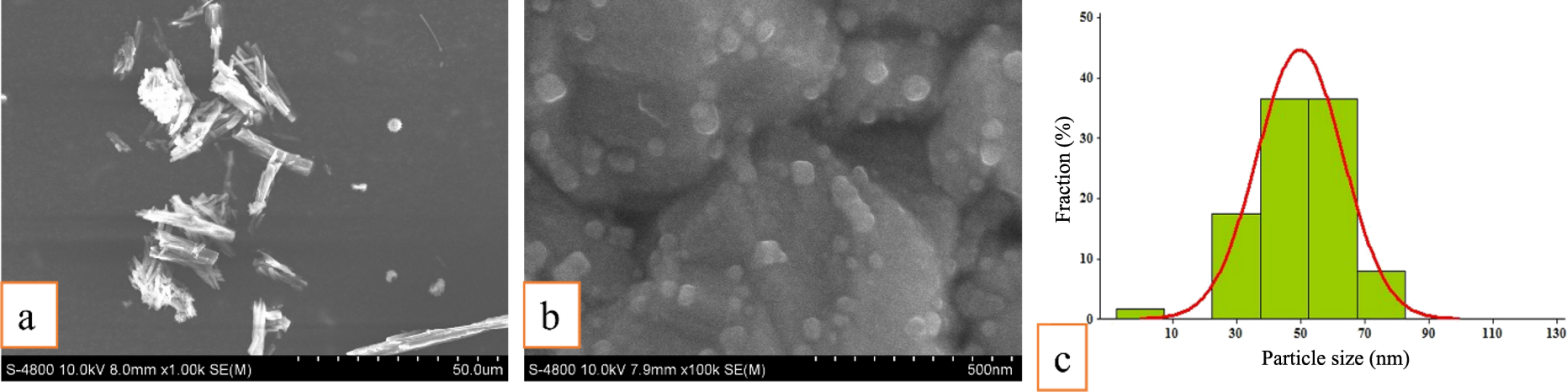
FE-SEM images of (a) berberine and (b) BerNPs. (c) Histogram of particle size distribution of BerNPs.

In this study, the raw berberine powder exhibited a crystalline structure with particle sizes ranging from 15 to 35 µm (Figure 1A). Ball milling is an efficient technique for fabricating nanoparticles from crystalline structures of organic pharmaceutical raw materials [18]. Other studies have shown that incorporating surfactants into the wet ball milling process significantly enhances particle size reduction, facilitates the effective production of nanoparticles, and preserves the sterility of the material [19–20]. In this study, sodium dodecyl sulfate, a negative ion surfactant, was adsorbed in the production process onto the surface of nanoparticles, resulting in negatively charged particles. Particles with the same surface charge repel each other, reducing the likelihood of agglomeration and deposition, and enhancing the stability of the suspension [21–22]. Consequently, a suspension of the BerNPs was produced with uniform particle sizes, predominantly ranging from 40 to 65 nm (Figure 1B,C). In our study, the rotary grinding method demonstrated superior efficiency in producing nanoscale berberine. Tran et al. employed the ball milling technique to produce BerNPs, achieving a particle size of 570.7 nm [23]. Piri et al. utilized the anti-solvent precipitation method, yielding BerNPs with an average particle size of 75 nm [24]. BerNPs were synthesized by ASP using glycerol as a safe organic solvent, resulting in BerNPs with a narrow size distribution and an average diameter of 156 nm [12]. Additionally, the high-pressure homogenization method reduced the average size of BerNPs to approximately 72.4 nm [25]. Numerous studies showed that smaller particle sizes results in a larger total surface area, thus significantly increasing biological activity and stability [20,28]. Compared to other studies, the size of the berberine nanoparticles obtained in this study was smaller, highlighting the significant application potential of the material.

**Figure 2:**
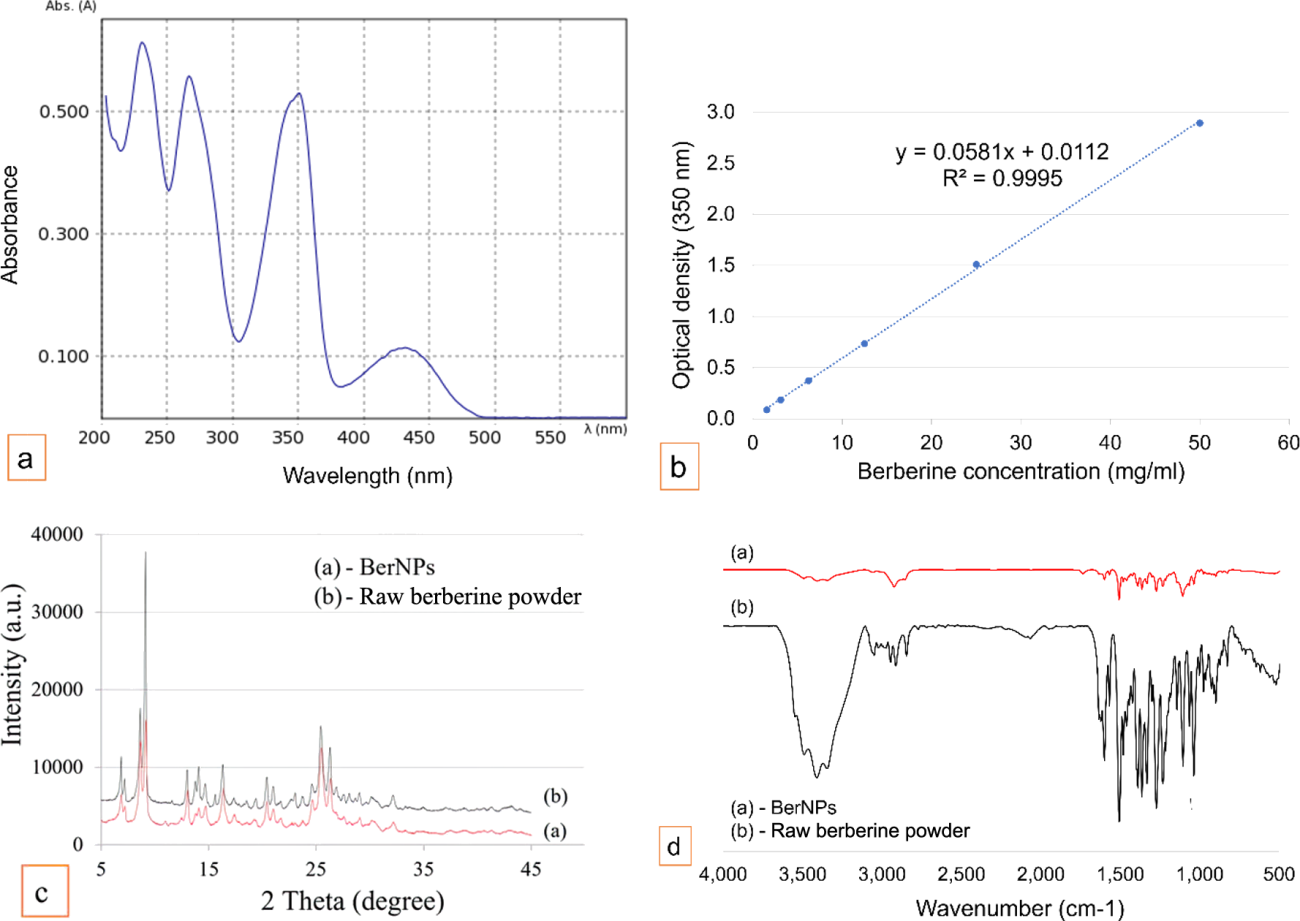
(a) UV–vis absorption spectrum of BerNPs. (b) Standard curve of pure berberine. (c) XRD patterns of pure berberine and BerNPs. (d) FTIR spectra of pure berberine and BerNPs.

UV–vis analysis of BerNPs samples revealed four characteristic absorption peaks of berberine at 230, 266, 350, and 430 nm (Figure 2A). These peaks are consistent with previous studies on the characteristic absorption spectra of berberine [26–27]. According to the standard curve (Figure 2B), the pure berberine content in the powder material was determined to be 92% by mass.

In both raw berberine powder and BerNPs, X-ray diffraction analysis showed strong peaks around 9.5° and weak peaks between 25.5° and 26.5° (Figure 2C). These characteristic diffraction peaks represent the crystalline structure of berberine [24,28], which shows that ball grinding does not alter the crystal structure of berberine. FTIR spectroscopy results show that the characteristic peaks of BerNPs coincide with that of the raw berberine at 1597, 1507, 1363, 1276, 1103, and 1035 cm^−1^ [24]. These results demonstrate that ball grinding has a purely physical impact, without altering the chemical structure or generating new chemical bonds.

### Antibacterial activity of BerNPs against *S. mutans*

**Figure 3:**
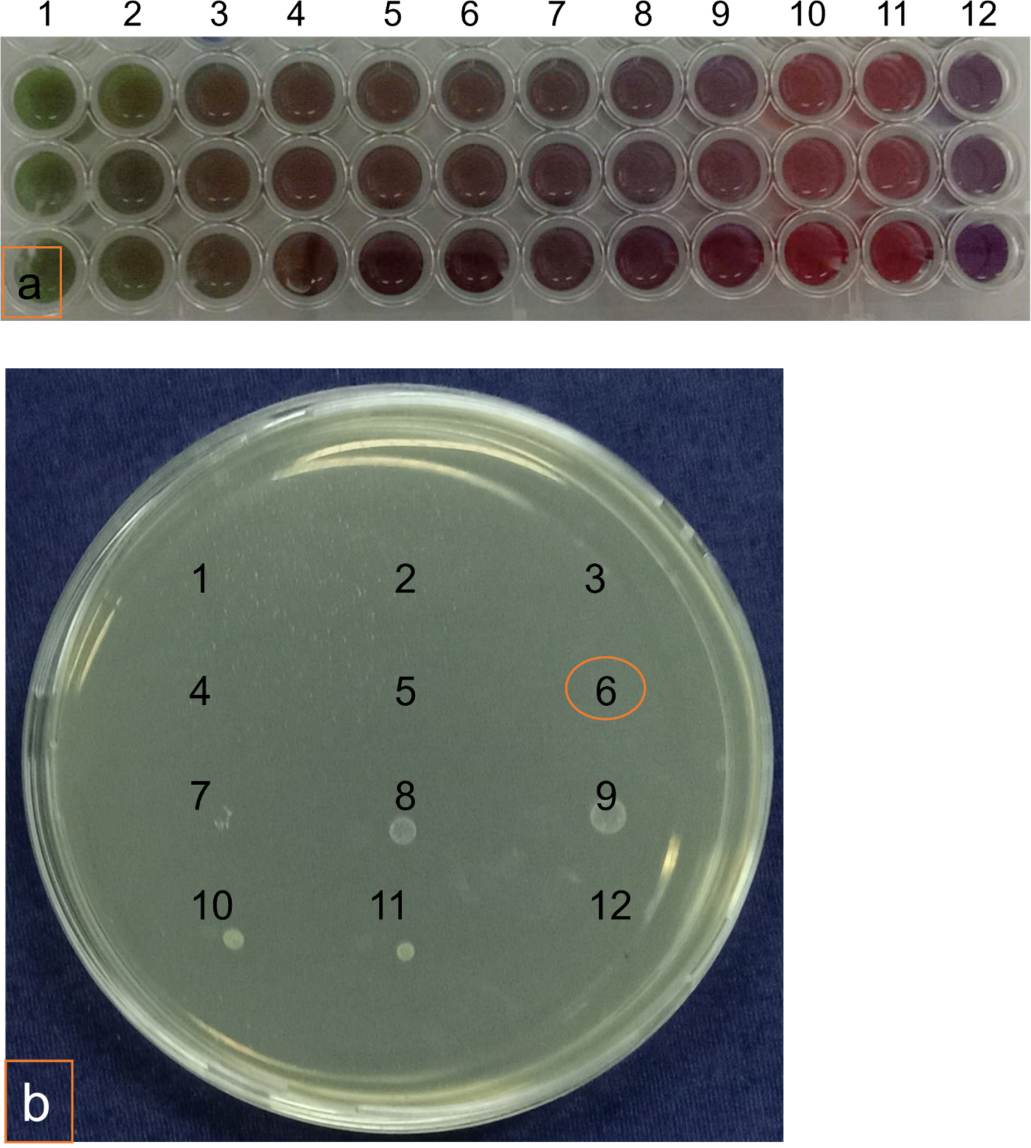
(a) Minimum inhibitory concentration values and (b) minimum bactericidal concentration values of BerNPs against *S. mutans*. Wells 1–10 contained two-fold serial dilutions of BerNPs (ranging from 5000.0 to 19.5 µg/mL) with 10^8^ cfu/mL of *S. mutans*, the eleventh well contained only 10^8^ cfu/mL of *S. mutans*, and the twelfth well contained only BerNP solution. The experiment was conducted in triplicate.

The minimum inhibitory concentration (MIC) was determined using the microdilution method. The lowest concentration at which the color of blue resazurin did not change was recorded as the MIC value. As shown in Figure 3A, the eigth well, corresponding to a concentration of 78.1 µg/mL, did not exhibit a color change. The minimum bactericidal concentration (MBC) value, illustrated in Figure 3B, was determined through the absence of colony formation on the agar plate, which corresponded to the sixth well at a concentration of 312.5 µg/mL. These results indicate that the MIC and MBC values of BerNPs against *S. mutans* are 78.1 and 312.5 µg/mL, respectively. In the study by Dziedzic et al., the antibacterial activity of berberine chloride against *S. mutans* was reported with a MIC of 1024 µg/mL and a MBC of 2054 µg/mL [29]. Antibacterial activities of berberine and BerNPs have been documented by various researchers. Nguyen et al. indicated that BerNPs prepared via the antisolvent precipitation method achieved MBC values of 2 and 5 mg/mL against methicillin-resistant *Staphylococcus aureus* (MRSA) and *Escherichia coli* O157:H7 strains, respectively [12]. Xia et al. assessed the antibacterial activity of berberine against MRSA, with MIC values ranging from 64 to 256 mg/L, depending on the bacterial subtype. Additionally, combining berberine with either clindamycin or rifampicin effectively reduced the bacterial density to one hundredth within 24 h [30].

The study by Sun et al. demonstrated that berberine produced notable antibacterial inhibition zones against four strains of *Cutibacterium acnes*, with MIC values ranging from 6.3 to 12.5 µg/mL and MBC values ranging from 12.5 to 25.0 µg/mL [31].

However, there is limited research on the activity of BerNPs against *S. mutans*, a primary pathogen responsible for dental caries. This study aims to provide additional information on the potential and applications of BerNPs in the development of oral care products.

### The effect of BerNPs on *S. mutans* cell membranes

**Figure 4:**
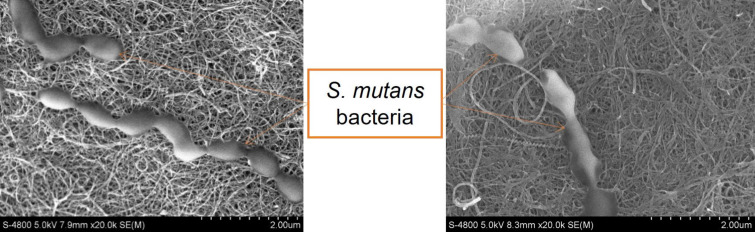
FE-SEM analysis. (a) *S. mutans* control without BerNPs. (b) *S. mutans* exposed to BerNPs at a concentration of 78.1 µg/mL.

FE-SEM analysis revealed that, under normal culture conditions, *S. mutans* displayed typical streptococcal morphology, characterized by spherical shapes arranged in chains or pairs. The cell surface appeared smooth, uniformly colored, and free of wrinkles (Figure 4A). However, after exposure to BerNPs, significant alterations in bacterial cell morphology were observed (Figure 4B). The cell membrane was severely compromised, exhibiting wrinkled and disrupted borders, leading to membrane rupture. This damage caused the release of intracellular substances, resulting in color fading and the emergence of graphene-based filaments.

Du et al. showed that berberine affects *Streptococcus pyogenes* by regulating proteins in the KEGG pathway, leading to the accumulation of reactive oxygen species (ROS) hindering the biosynthesis of DNA, proteins, and lipids, ultimately triggering cell death [32]. The findings of Peng et al. on *Streptococcus agalactiae* indicated that berberine significantly disrupted the cell membrane structure. SDS-PAGE electrophoresis results showed that some protein bands were blurred or absent, suggesting that berberine led to complete or partial degradation of the proteins [33]. In this study, FE-SEM analysis further confirmed that one of the mechanisms by which BerNPs kill *S. mutans* involves the disruption and damage of the bacterial membrane.

### Inhibition of biofilm formation

**Figure 5:**
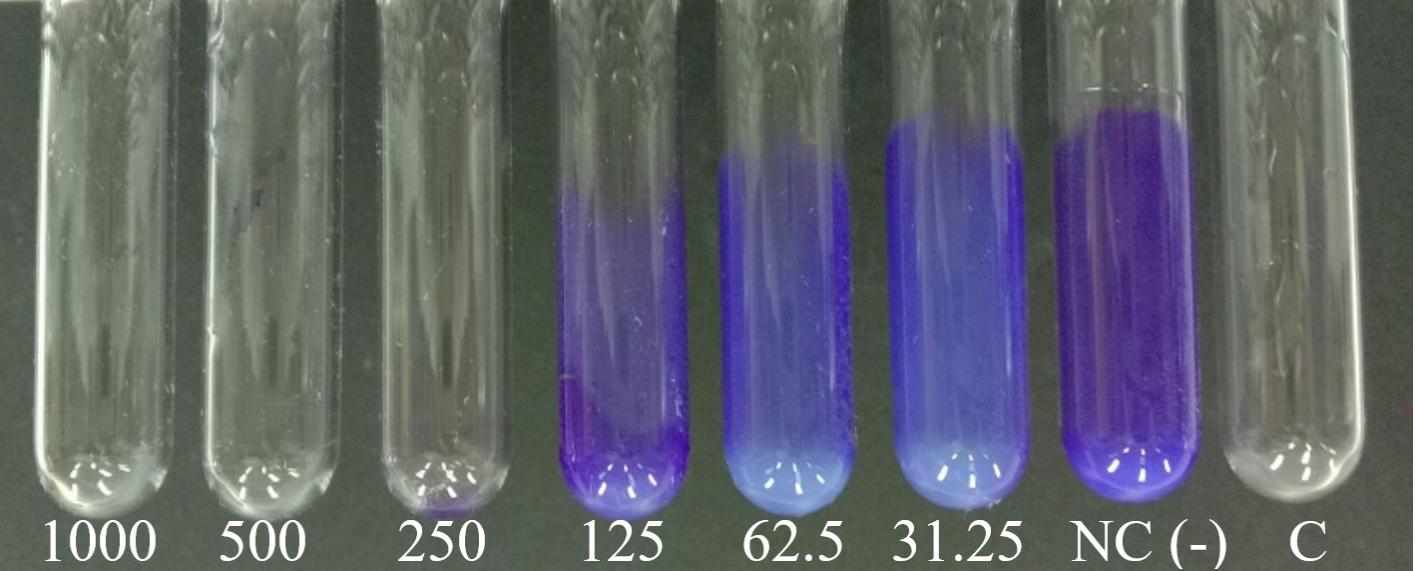
The ability of BerNPs to inhibit biofilm formation by concentration of BerNPs. The numbers represent the corresponding concentrations in µg/mL.

*Streptococcus mutans* is the primary cause of dental caries [34]. It contributes to tooth damage through two main mechanisms, namely, dental erosion due to acid production and the formation of biofilms that produce plaque on teeth [35]. Berberine has been reported to inhibit biofilm formation of *Candida albicans* and *Staphylococcus aureus* [36–37]. In this study, BerNPs demonstrated the ability to inhibit biofilm formation at various concentrations (Figure 5). The biofilm formation of *S. mutans* relies on the secretion of glycosyltransferase enzymes and several membrane-bound proteins [38]. SrtA, a surface protein involved in adhesion, biofilm formation, and biosynthesis of *S. mutans*, has been reported to be inhibited by berberine [39]. The inhibition of biofilm formation by *S. mutans* was also observed in the study by Zhou and coworkers. The results showed that berberine chloride hydrate effectively downregulated the expression of the genes *srtA*, *spaP*, *gbpC*, *comX*, and *ldh*, thereby preventing biofilm development [40].

## Conclusion

In this study, BerNPs were fabricated using ball milling with zirconium balls. Analysis through FE-SEM, UV–vis, XRD, and FTIR revealed that the nanoparticles predominantly exhibited a crystalline structure, with an average size of 40–65 nm. Biological activity tests demonstrated that BerNPs effectively inhibited the growth of *S. mutans*, with MIC and MBC values recorded of 78.1 and 312.5 µg/mL, respectively. FE-SEM analysis further indicated that BerNPs caused lysis of *S. mutans* cells by targeting the cell membrane. Additionally, BerNPs were shown to inhibit biofilm formation, a key factor in tooth decay caused by *S. mutans*. These findings provide a foundation for developing products containing BerNPs for use in dental care and the treatment of oral health issues.

## Materials and Methods

### Materials

*Streptococcus mutans* ATCC 35668^TM^ (Microbiologics, USA) was acquired from the Biotechnology Laboratory, Center for Research and Development of the Hi-Tech Park in Ho Chi Minh City, Vietnam. Bacteria were cultured at 37 °C in tryptone soya broth (TSB) medium (Scharlau, Spain) for 24 h. Raw berberine powder (pharmaceutical standard) was sourced from Novaco Pharmaceutical Joint Stock Company Ho Chi Minh City branch (Ho Chi Minh City, Vietnam).

### Production of BerNPs

BerNPs were fabricated through wet-milling with zirconium balls. The suspension was prepared with 4 g of berberine, 0.75 g tween 80 (Scharlau, Spain), and 0.25 g of sodium dodecyl sulfate (Scharlau, Spain), and filled up to a total weight of 100 g with sterile distilled water. Zirconium balls (200 g) were added to the vial, which was placed on a platen roller and operated at 2,000 rpm for 120 h. After milling, the balls were removed, and the suspension of BerNPs was collected. Powder BerNPs were obtained by the freeze-drying for 48 h at −55 °C under vacuum.

### Physicochemical characterization of BerNPs

The specific adsorption peak of BerNPs was determined by UV–vis absorption spectroscopy in a wavelength range from 200 to 600 nm after dilution in methanol. The berberine concentration in BerNPs was determined based on a berberine powder standard curve (SIGMA HPLC standard) at a wavelength of 350 nm.

FE-SEM (Hitachi S-4800, Japan) was employed to determine the form and particle size of BerNPs. A small sample was placed on a copper net (Sigma-Aldrich, USA), dried at room temperature, and analyzed at a voltage of 10 kV [24].

X-ray diffraction analysis was used to evaluate the crystalline structure of berberine and BerNPs. FTIR spectra were analyzed to identify typical functional groups and chemical bonds in raw berberine and BerNPs [28].

### Determination of minimum inhibitory concentration

The MIC value was determined according to the modified method of Solanki and coworkers [41]. The stock solution of BerNPs was prepared at a concentration of 10,000 μg/mL. Next, 100 µL of TSB was added to each well of a 96-well plate. Subsequently, 100 μL of BerNPs was added to the first well, and two-fold serial dilutions were performed up to the tenth well, resulting in concentrations ranging from 5000.0 to 19.5 µg/mL. The two negative control wells, the eleventh and the twelfth, contained only the bacteria solution and only BerNPs solution, respectively. Following this, each well (excluding the twelfth) was inoculated with 100 µL of *S. mutans* solution at a density of 10^8^ cfu/mL. The plates were then incubated for 24 h at 37 °C under anaerobic conditions. The experiment was conducted in triplicate. After incubation, 60 µL of 1× resazurin was added to each well, and the plate was incubated for an additional 30 min. The MIC value was determined as the lowest concentration at which the blue color of resazurin remained unchanged.

### Determination the minimum bactericidal concentration

The MBC values were determined according to the protocol of Mah and coworkers [42]. 3 µL of the suspension from the wells in the MIC assay were dripped on the TSA plate and incubated at 37 °C for 24 h. The MBC value was determined as the lowest concentration at which no visible bacterial colonies were observed.

### Evaluation of the effect of BerNPs on *S. mutans* cells

Filed-emission scanning electron microscopy (FE-SEM) was used to visualize the impact of BerNPs on the cell walls of *S. mutans* [21]. A BerNPs solution at the MIC was prepared by diluting in TSB medium. *S. mutans* (final concentration of 10^8^ CFU/mL) was then added to the BerNPs solution. Negative control tubes contained only bacteria. All test tubes were incubated at 37 °C. After 24 h, the samples were fixed onto a graphite substrate, and FE-SEM imaging was performed.

### Inhibition of biofilm formation

The Crystal Violet Biofilm Assay was used to evaluate the biofilm formation of *S. mutans* [43]. BerNPs solution was diluted in TSB + 1% sucrose medium. *S. mutans* inoculum was added to the test tubes to achieve a final concentration of 10^8^ cfu/mL (3 mL in total volume). The positive control was prepared without BerNPs, and the negative control contained only TSB + 1% medium sucrose. All tubes were incubated at 37 °C. After 24 h, all the solutions were removed from the tubes, and the test tubes were washed twice with sterile distilled water. Then, 3 mL of 1% crystal violet (Merck, Germany) was added to each tube. After 20 min, the dye solution was discarded, and the tubes were washed twice with sterile distilled water. Biofilm formation was recorded by observing the stained cells attached to the walls of the test tubes.

## Data Availability

All data that supports the findings of this study is available in the published article and/or the supporting information of this article.
